# American Academy of Dental Science

**Published:** 1894-08

**Authors:** 

**Affiliations:** American Academy of Dental Science


### Reports of Societv Meetings.

### AMERICAN ACADEMY OF DENTAL SCIENCE.

The regular monthly meeting of the American Academy of Dental Science was held at Young's Hotel, Boston, on Wednesday evening, April 4, at six o'clock, President Smith in the chair.

A paper by Dr. J. W. Farlow—subject, "On some Relations of Diseases of the Nose and Throat to Dentistry"—was then read.

(For Dr. Farlow's paper, see page 491.)

### DISCUSSION.

*Dr. Eames.*—I probably express the gratitude of all to the speaker of the evening for outlining in so simple and clear a manner the symptoms of adenoid disease.

My interest in this and allied subjects arises from the dental stand-point. I have endeavored to cover a field not wholly covered by the physician or the dentist,—a middle ground.

I am interested to find out the cause of this disease, and of the relation of these adenoid growths to deformities of the dental arch and teeth. I may give it as my decided belief that the adenoid growth does not produce the deformed palate, but that they are concomitants and have a common cause. I have two patients, who came to me at nearly the same time, with adenoid disease, and I should be pleased at any time to show you models of the cases, or the patients. One of them has irregular teeth and a contracted arch, while the other has what most of us would call a typical arch, wide, well formed, with teeth regular, but with more adenoid disease than the first case. These two cases represent extremes, as far as the palatal arch is concerned. It has been said that it is useless to correct irregularities of the teeth while there are adenoids present; but I have never seen proof of this, and I do not believe it is true. I have seen one case, at least, in which the arch was expanded and teeth regulated before it was known that adenoids existed, and three years afterwards, when the growths were removed, there had been no return to the former condition. While I believe that the growths should be removed, and that at the earliest opportunity, I do not believe that the presence of adenoids and consequent mouth-breathing produce a contracted arch.

*Dr. Fillebrown.*—I would like to ask Dr. Farlow in how large a proportion of cases does the high arch accompany the adenoids? Or, to put it the other way, how frequently do you find adenoids with a normal arch?

*Dr. Farlow.*—Very infrequently, except in adults, and that to a limited extent. It is possible that adenoid disease can begin at a time when the jaw has very nearly reached its full growth, and in such cases the teeth, holding their positions firmly, often deceive one into thinking that there is no adenoid present. I have a young lady patient who is a good example of this class. She has a well-formed arch and a beautiful set of teeth, and yet there is considerable adenoid growth present, apparently starting from either measles or scarlet fever, or some other infantile disease which she did not contract until later than usual. This disease, whatever it was, produced some affection of the post-nasal space, and now the adenoid has become added to it. I think there are a number of such cases, but I should say that probably in seventy-five per cent. of the cases that I operate on in young children, where there is an obstructive growth in the post-nasal space, there is also a high palate, looking like the imprint of a finger on putty.

*President Smith.*—With great narrowness between the bicuspid teeth and canines and overlapping of the incisors?

*Dr. Farlow.*—Yes.

*Dr. Fillebrown.*—What led me to ask my last question was the case of a young girl which came under my observation not long since. The only troublesome symptoms which she showed were frequent colds and a little obstruction in speaking. The arch was normal, the bicuspids being a little approximated, but not enough to need any attention. Examination proved the presence of adenoids. The patient had complained somewhat of difficulty of breathing through the nose, and the removal of the adenoids of course improved the respiration and helped the other symptoms that I speak of.

*Dr. Farlow.*—I believe there are some people who can overcome almost any obstruction. I have a young lady patient with rosy cheeks who is the picture of health, but the amount of adenoid growth is very considerable, and shows itself by very frequent colds and profuse secretion. We should bear in mind the size of the growth and the size of the space in which it is contained. It makes a great difference to the patient whether it is a small growth in a large place or a large, secreting, easily swelling growth in a place barely large enough to contain it.

Very often the growth atrophies at puberty, and the disease in adults is of a firmer, harder character than in children.

I have seen a few cases of high palate in children where no adenoid disease existed, and also a few cases of adenoid disease without the high palate, but in the vast majority of cases the two are associated, making it extremely probable that the deformity of the palate is caused by the nasal obstruction.

By many this deformity is attributed to thumb-sucking, but in the marked cases the nose is so obstructed that it is impossible to suck the thumb for any length of time.

*President Smith.*—The deformity produced in the jaw by the adenoid growth and that produced by thumb-sucking are entirely different. Thumb-sucking never produces a V-shaped arch.

*Dr. Farlow.*—There are men in your profession who think differently.

*President Smith.*—I know it, but they are wrong,—they confuse the two. With a V-shaped arch mesial surfaces of the central incisors come to a point like a plough, whereas in thumb-sucking or arm-sucking, the teeth span out. The two central incisors point out, giving the alveolar processes in front an inclination to spread rather than to come to a point, and forming a broad arch such as Dr. Eames has spoken of.

*Dr. Fillebrown.*—There is one more question I want to ask Dr. Farlow, and that is, How generally is the V-shaped arch dependent upon the presence of adenoids? In other words, how large a percentage of V-shaped arches do you find where there are no adenoids?

*Dr. Farlow.*—Very few, indeed. The only cases which I should be likely to see would be those who are troubled with some affection of the throat, and many cases must exist which do not come to the doctor's attention. But I see quite a number of cases where the palate is high and where there is a certain amount of irregularity of the tissue at the vault. Such are cases where adenoid disease has formerly existed and later has atrophied, so that they do not belong to the category of V-shaped palate independent of adenoid disease.

It is possible for the post-nasal hypertrophy to start up in young adult life and after the teeth and jaws are well developed, in which case the palate might not be deformed; but I should say that in more than seventy-five per cent. of the children on whom I have operated the palate was either somewhat V-shaped or high.

I have a patient on whom I am to operate in a few days. He has a broad flat arch, like the case that Dr. Eames spoke of. I found that he could breathe freely through the nose, and supposed, of course, there was no special post-nasal hypertrophy. Instead, however, there was quite a large secreting mass at the vault. One of his nostrils was very large, as was also the post-nasal space, so that he had good nasal respiration, even with a large amount of adenoid growth.

*Dr. Banfield.*—Would Dr. Farlow expect that in the case of a child exhibiting a catarrhal condition the removal of the adenoids would do away with it?

*Dr. Farlow.*—Yes; in very many cases. But the nose may also need treatment, and some of the worst forms of catarrh are associated with a condition of atrophy of the nose rather than hypertrophy, and have no connection with adenoid disease.

*Dr. Banfield.*—But may not that catarrhal condition be entirely due to the adenoid?

*Dr. Farlow.*—It certainly may, and removal of the post-nasal obstruction may rid the child of what was keeping the whole nose in an inflamed, congested, and secreting condition, and the establishment of ventilation and drainage may restore the nose to health.

*Dr. Williams.*—There is a point bearing on Dr. Farlow's idea which I have heard suggested, and that is with regard to the effect of mouth-breathing in connection with adenoid disease in producing the V-shaped arch. The theory is that the deformity is partly due to the pressure of the muscles of the cheek. That is a theory which I think is very reasonable. When one opens the mouth widely he will notice a considerable strain on the muscles, and very likely that constant pressure in mouth-breathers would alter the shape of the jaw.

*Dr. Eames.*—I should most heartily dissent from the statement that the open mouth contracts the palatine arch, or that adenoid growths do it. I do believe, however, as has been said, that these are often found together; that in cases of contracted arch there is also usually a deformity of the nasal septum, and other deformities, but I do not believe that the mouth opened sufficiently wide to admit air for breathing presses upon the upper arch so as to draw it together. I think I can show by experiment at some future time just how much pressure is brought to bear on the side teeth by opening of the mouth.

*Dr. Williams.*—The condition is frequently illustrated in patients who find it difficult to brush the sides of the upper teeth owing to tenseness in muscles of the cheek. If the cheek is so tense as to prevent the brush from passing along the sides of the teeth, it is reasonable to suppose that, if put to an extra strain by the dropping of the jaw, this tenseness could gradually cause contraction of the jaw. Then, again, see how slight a pressure is required to move teeth. You see strong, firmly set teeth moved almost at will by a little wedge of inflamed gum.

*Dr. Eames.*—I would like to ask if it is not the ramus of the jaw that prevents a large tooth-brush, perhaps half an inch wide, from passing up along the sides of the teeth?

*Dr. Williams.*—Sometimes; but very often it is the pressure of the cheek against the teeth.

*Dr. Fillebrown.*—I think the obstruction you speak of is from the voluntary contraction of the muscles.

*Dr. Williams.*—No; it is from the simple dropping of the jaw, though the result is practically like a voluntary contraction.

*Dr. Eames.*—If you will just watch children and others when they are asleep with their mouths open, you will see that they open the mouth only a very little; the mouth simply drops open. There is not a tense condition of the muscles.

*Dr. Farlow.*—Many of the sufferers from adenoid disease have large tonsils also, and during sleep the tongue drops back and the mouth is, of necessity, widely opened, so that it is not simply a case of relaxation of the jaws, but the effort to breathe and the snoring are very violent and can be heard throughout several stories of the house.

*President Smith.*—I am inclined to agree with Dr. Williams that the tension of the cheek muscles may have an influence in contracting the arch in early childhood, when the alveolar process of the upper jaw is soft. Of course we don't know how much resistance the bones of the upper jaw have, but we know this, that many children when they go to bed have a habit of simply opening the mouth and putting in their thumb or closing their mouth on their arm; there is no great force there, they merely rest their teeth in that position. Now, I know of a case where the incisors and alveolar process stand out very prominently, and it was caused by arm-sucking. There have been cases of deformities produced by children sucking their thumbs for only a short time, but long enough to produce a slight alteration in the line of the arch before the eruption of the teeth, and the teeth erupted in the abnormal line. Now, if such slight things as these produce deformities, it seems to me that extensive mouth-breathing would produce what we call the V-shaped arch. All V-shaped arches are not so caused, but the causes mentioned tend to produce this shape.

*Dr. Ainsworth.*—My experience in this line leads me to think that mouth-breathing is very largely the cause of narrow arches. The slight pressure caused by the strain of some of the forward muscles, as the orbicularis oris, rather than of the masseter or the buccinator muscles, is responsible for this.

There is another point which has not been referred to in describing the cause of the upper teeth not taking their proper position, and that is the lack of occlusion with the under teeth. When the mouth is closed the interlocking of the cusps guides the upper teeth into place, and that influence is largely lost where mouth-breathing exists to a considerable extent. I am very strongly inclined to side with the views of Dr. Williams and Dr. Smith.

*Dr. Banfield.*—Speaking of snoring, you know that is not entirely confined to children, and that older persons sometimes succeed in making themselves obnoxious to others without knowing it. I would like to ask if, in those cases, you would expect to find some growth as the cause of it?

*Dr. Farlow.*—No, indeed. That is not, by any means, the only cause of snoring. It may be due to a weak relaxed palate, or to obstruction in the nose itself, as from turbinated bones or deviations of the septum; or it may exist where there is no obstruction to nasal respiration. Adenoid disease is not at all common after thirty to thirty-five years of age.

*Dr. Briggs.*—It seems to me that we do not put enough weight on the general vitality of the patient with reference to this obstruction, and that the results may not always express themselves in the same locality. If a patient has enough surplus vitality, he may overcome a great many of the ordinary symptoms; but if he has not surplus vitality, the result is bound to show itself somewhere,—it may not be in the mouth and jaws, it may be in the back. A child who does not breathe through the nose does not inflate the lungs to their fullest capacity; the lungs cannot be filled as full through the mouth as through the nose, and, therefore, all these things tend to affect the general development of the child, and are likely to result in some deformity. Where that malformation is depends upon circumstances, but you could expect in a child of poor vitality to find some evidence of poor development in some part of the body. I have a case in mind where adenoid disease has existed. The patient has no malformation about his mouth, has a good arch and a good palate, for at that particular point the natural development has established itself in spite of the adverse conditions. And yet, who is prepared to say that the double curvature which this patient has been suffering from may not be in part due to the impaired vitality resulting from the presence of the adenoid obstruction, as well as to the impairment of his vitality through his digestion, because of the immense mucous discharge that has been pouring down into his stomach.

*Dr. Banfield.*—I would like to ask Dr. Farlow if he finds among children who have adenoid growths a more irritable or nervous condition? I have thought that they were more nervous and more liable to certain forms of disease, such as colds, indigestion, or some diseases of a more nervous nature.

*Dr. Farlow.*—I have seen a large number of nervous phenomena caused by adenoid hypertrophy, such as chorea, twitching, general irritability, and one case of convulsions. If we pass a probe to the top of the post-nasal space, we often see reflex symptoms manifest themselves in various directions, as from this region nerves are given off which go to many and important structures.

I have seen cases where gentlemen of your profession have undertaken to straighten the teeth without removing the adenoids, and the children have been made so nervous that the dentists were unable to continue their work. You remember the case, Dr. Briggs, of the Barker boy and sister, who became very nervous while wearing regulating appliances. That was a very marked case; any attempt at operation in the chair rendered them almost uncontrollable, producing a decided rise in temperature. After I had removed the adenoids and cleared the post-nasal space, Dr. Briggs was able to continue and to get a good jaw. I do not quite agree with Dr. Eames about that part of it.

*Dr. Eames.*—I agree fully with Dr. Farlow as to the necessity of removing the adenoids. It was not that the operator would not do all that he has said, or that I do not fully recommend such an operation; but I simply stated it as my belief that the presence of adenoids would not cause the return of any irregularity which had been corrected before their removal.

*Dr. Briggs.*—It seems to me that the point of impairment of vitality is not fully appreciated. I think Dr. Farlow will bear me out in saying that it has a great deal to do with the expression of symptoms. Every person whose health is impaired does not have the same expression of this impairment,—one has rheumatism, another has a cold in the head, and so on. And so in every child whose vitality is impaired the same conditions do not obtain. It all depends, then, upon the impaired condition of the general health of the patient, whether he exhibits the more pronounced symptoms of adenoid disease, or whether he simply does not breathe in a normal manner. We ought to know where the child is upset, for there may be matters of the general condition which may be changed almost instantly by the removal of these adenoid growths.

As to the statement that the teeth would return after correction while the adenoids were present, I think that would depend a good deal upon the development of the patient. If he is matured and you correct the teeth, even if you do not correct the adenoids, of course the teeth would remain as you left them. I do not think one would be expected to reason, as far as the adenoids are concerned, that the teeth would return if the operation were performed at that stage of development. The question comes, in the earlier years, when everything is soft and pliable, then you would not want to regulate before the adenoids were removed, as the same causes operating on teeth which have not yet reached a reasonable degree of firmness would be likely to produce the same results.

(Subject passed.)

*President Smith.*—Gentlemen, I now have the pleasure of introducing to you Dr. Douglas Graham, who will read a paper entitled

"Massage in Rheumatism of the Temporo-Maxillary Articulation and Muscles of the Lower Jaw."

(For Dr, Graham's paper, see page 487.)

DISCUSSION.

*Dr. Briggs.*—I am a great believer in massage, and I have had it employed in my family more or less with great benefit. The question of its application to the jaw has come to my notice in some cases with considerable benefit to the patient. I know that all of you have met with patients whose jaws snap in eating sometimes so bad as to be quite annoying to others at the table, and in the few cases that have come to me in my practice there has been an improvement, if not cure, by recommending such a person to go to a masseur or to massé the jaw himself. In that way the ligaments and the muscles have been toned up and improved. I have also had it suggested to me—I have never tried it yet—that a proper masséing of the gums, probably by the patient himself, was of great benefit in cases of passive congestion of the gums. For instance, in that sort of condition which we find in pyorrhœa alveolaris, it seems to produce a proper establishment of the circulation about the parts, and, therefore, an improvement of the general condition of the gums.

*Dr. Williams.*—For some years I have been in the habit of directing patients who complain of looseness or lameness of the jaw, sometimes snapping, sometimes not, but with difficulty of closing it regularly, caused by laxity in either the ligaments or muscles, or both, to take twice a day, morning and night, a towel wet in cold water and slap the face, and then take a dry portion of the towel and rub it briskly. That is, perhaps, combining the shower-bath with massage.

*Dr. Clapp.*—I would like to ask Dr. Graham if he thinks massage would be of any service in the acute stages of alveolar abscess. Dr. Briggs spoke of it in rather a different sense, but I was about to ask this question before Dr. Briggs spoke. In the early stages of threatened abscess, would this treatment assist in the distribution of the blood and thereby abort an abscess?

*Dr. Graham.*—I am afraid I do not know enough about dentistry to give a definite answer to that question; but in other parts of the body massage will sometimes abort the commencing inflammation about the joints, and if it were possible to apply it to the gum so as to bring about a better circulation, I do not see why it would not do it there.

*Dr. Banfield.*—I would agree with Dr. Briggs in the efficacy of massage in pyorrhœa. In a number of cases where I have advised treatment of this kind, there has been marked improvement and decrease in the amount of inflammation.

I would like to inquire of Dr. Graham if he thinks that this pain which patients complain of in the location of the maxillary articulation is due to a rheumatic condition? I have had a number of patients for whom it has been difficult to operate on account of great pain at the temporo-maxillary articulation. Are loose-jointed persons more apt to have rheumatic affections than others?

*Dr. Graham.*—One could not make out a clear diagnosis unless he got some symptoms of rheumatism in other parts of the body. I think loose-jointed people are more subject to rheumatic affections than others.

*Dr. Banfield.*—Does this pain that patients complain of in the articulating region come from the muscles or from the maxillary articulation?

*Dr. Graham.*—It may be either. I think one might be able to locate it, or get the patients to do so, either in the articulation or the muscles. They could probably tell you exactly where it is.

*Dr. Briggs.*—I would like to speak on the matter to which Dr. Banfield has referred, because I have treated it in a different way. I don't know that it is exactly a case for massage, except to relieve the pain for the time being. I have usually attributed it to ordinary cramp from the exercise of the muscles. I think you will find in cases where patients have so suffered that they, in a misguided way, have forced their mouths open to allow the dentist to operate, and it brings about muscular cramp; whereas, if a patient simply lets the mouth drop open, there is no extra tension on the muscles. I have a patient in mind. She is an adult person who had always suffered intensely when with the dentist in opening her mouth, and she now has no trouble, because I impress upon her the fact that she must not force her mouth open, but simply allow it to drop open, and that relieves her trouble entirely.

*Dr. Banfield.*—A few years ago I had a patient, a young man, whose jaw would slip out of its sockets from very slight pressure. While operating upon his teeth I would handle his jaw carefully and instruct him not to open it more than necessary; but occasionally he would find himself unable to close his jaw and would point to it for me to replace. He said he was obliged to deny himself a good hearty laugh, and was especially careful when in society.

*Dr. Williams.*—Was it a full dislocation?

*Dr. Banfield.*—Yes. The dislocation did not throw the jaw to one side, therefore it must have been a full dislocation.

*Dr. Williams.*—Was the patient in the habit of opening his mouth forcibly, or did he let his mouth drop open?

*Dr. Banfield.*—He tried to open it as naturally as possible, and did not open it more than was necessary.

*Dr. Williams.*—I say to my patients, "Let your jaw drop naturally, as if you were sleeping with your mouth open; do not make any effort." And I very seldom have any one complain of pain from opening the mouth.

*Dr. Potter.*—I would like to ask Dr. Graham about this snapping of the joints, whether he has used for it electricity,—the interrupted current,—and how that compares with massage? I know of its having been used in one case with a great deal of satisfaction.

*Dr. Graham.*—The interrupted current combined with massage works very nicely, and is better than either one alone. This current will relieve the pain so that patients snap the joints as much as they want to and think it great fun, and it does effect a cure.

William H. Potter, D.M.D.,
*Editor American Academy of Dental Science.*

